# The provenance of the stones in the Menga dolmen reveals one of the greatest engineering feats of the Neolithic

**DOI:** 10.1038/s41598-023-47423-y

**Published:** 2023-12-01

**Authors:** José Antonio Lozano Rodríguez, Leonardo García Sanjuán, Antonio M. Álvarez-Valero, Francisco Jiménez-Espejo, Jesús María Arrieta, Eugenio Fraile-Nuez, Raquel Montero Artús, Giuseppe Cultrone, Fernando Alonso Muñoz-Carballeda, Francisco Martínez-Sevilla

**Affiliations:** 1 Canary Islands Oceanographic Center (COC), Spanish Institute of Oceanography (IEO), Spanish Research Council (CSIC), Santa Cruz de Tenerife, Spain; 2 Department of History and Philosophy, Area of Prehistory, University of Alcalá (UAH), Alcalá de Henares, Spain; 3 Department of Prehistory and Archaeology, University of Seville (US), Seville, Spain; 4 Department of Geology, University of Salamanca (USAL), Salamanca, Spain; 5 Andalusian Institute of Earth Sciences (IACT), Spanish Research Council-University of Granada (CSIC-UGR), Armilla, Granada, Spain; 6 Department of Mineralogy and Petrology, Facultad de Ciencias, University of Granada (UGR), Granada, Spain

**Keywords:** Archaeology, Social anthropology

## Abstract

The technical and intellectual capabilities of past societies are reflected in the monuments they were able to build. Tracking the provenance of the stones utilised to build prehistoric megalithic monuments, through geological studies, is of utmost interest for interpreting ancient architectures as well as to contribute to their protection. According to the scarce information available, most stones used in European prehistoric megaliths originate from locations near the construction sites, which would have made transport easier. The Menga dolmen (Antequera, Malaga, Spain), listed in UNESCO World Heritage since July 2016, was designed and built with stones weighting up to nearly 150 tons, thus becoming the most colossal stone monument built in its time in Europe (c. 3800–3600 BC). Our study (based on high-resolution geological mapping as well as petrographic and stratigraphic analyses) reveals key geological and archaeological evidence to establish the precise provenance of the massive stones used in the construction of this monument. These stones are mostly calcarenites, a poorly cemented detrital sedimentary rock comparable to those known as 'soft stones' in modern civil engineering. They were quarried from a rocky outcrop located at a distance of approximately 1 km. In this study, it can be inferred the use of soft stone in Menga reveals the human application of new wood and stone technologies enabling the construction of a monument of unprecedented magnitude and complexity.

## Introduction

The geological characterisation and provenance of stones used for the construction of megaliths is of great value to understand the cultural and technical ability of prehistoric societies. These studies provide a great deal of technical information concerning the stone used in the architecture, as well as the techniques applied to quarry and transport them. Recent studies carried out at major world megalithic sites such as Stonehenge in Great Britain (e.g.,^1,2^), Valencina, in Spain (e.g.,^3–5^) or Easter Island (e.g.,^6,7^), show how geoarchaeological approaches based on petrology and geotechnics provide crucial data to understand the role of stone materials in producing monumental landscapes, involving aspects such as place-making, place-keeping and identity-building.

Although thousands of megaliths have been found in Iberia, geoarchaeological approaches have only been applied to a few of them. Apart from Valencina, mentioned above, such studies are available for Chabola de la Hechicera and other dolmens in Northern Iberia (e.g.,^8–10^); Vale Rodrigo and Anta da Lajinha, in the west^11–13^; Freixo-Redondo in the southwest^14^; Puigseslloses, in the north-east^15^; El Portillo in inner Iberia^16^ as well as Alberite^17^, Palacio III^18^, El Pozuelo^19^, and Panoría^20^ in the south. Albeit short, this list of studies reflects a growing interest in the subject and its potential for innovation in the analysis of late prehistoric monumentality.

Menga represents an excellent example to study the emplacement and construction of monuments among Neolithic societies. Menga is part of the Antequera dolmens site (Malaga, Spain) site, listed as a UNESCO World Heritage Site since July 2016 (Supplementary Text S1). Menga was studied for the first time in the 1840s^21^ and achieved worldwide interest throughout the 19th century as one of the earliest references for the study of the megalithic phenomenon (e.g.,^22^). Since the early 2010s this dolmen has been the focus of a renewed research efforts that have led to major discoveries and new insights regarding its cultural and social context during the first half of the 4th millennium BC^23–25^, and its subsequent long and complex biography as a monument^26–29^. Other features such as the lithologies of the massive capstones, the precise characterization of the quarry source and travelled distances remained unknown.

The Megalithic complex of Antequera is located in one of the enclaves with the most abiotic resources in the Prehistory of the south of the Iberian Peninsula^25^ (Figs. S1, S2). Menga is located in a gentle hilltop facing the Guadalhorce river lowlands. This hill vas frequented prior to the construction of the great dolmen. Locationally, this is the only position from which its chamber can face both La Peña de los Enamorados (The Lovers’ Rock) and sunrise at the same time^25^ (Fig. S3a,b). Megaiths are characterized by being made up of large stones (Fig. S3). Other features such as the lithologies of the massive capstones, the precise characterization of the quarry and travelled distances remained unknown until now.

We present the integrated results of fieldwork and laboratory analyses carried out over a decade, revealing the precise provenance of the massive stones used to build Menga. Our research is based on three main objectives: (i) petrological identification of each of the dolmen’s structural components and their sedimentological facies; (ii) lithological comparison of the materials described in the dolmen, with the marine facies for the Upper Tortonian (the Tortonian has an absolute age between 11,608 ± 0.005 and 7246 ± 0.005 Ma) of the Antequera zone; (iii) detailed geological cartography of the region surroundings.

## Results

### Lithological characterization of the Menga stones

**Figure 1:**
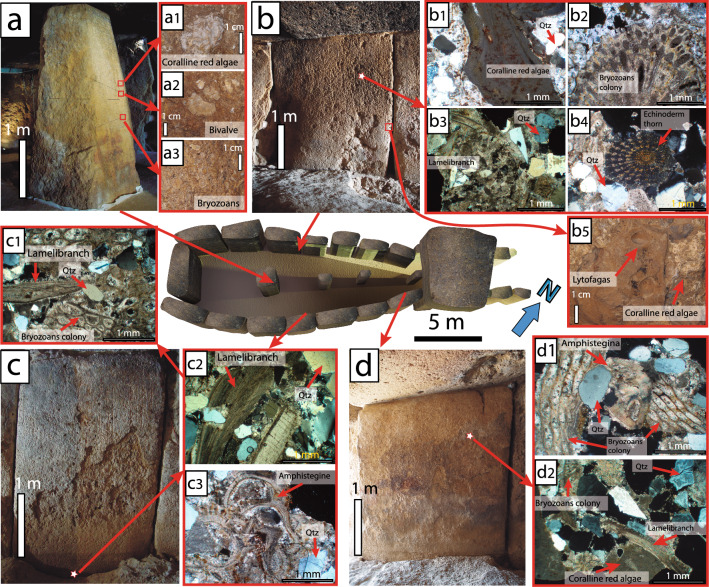
3D Model of Menga drawn with AutoCAD showing the biofacies (microfacies) present in the stones. The fourth pillar, currently missing, has been added, while capstones C-2, C-3, C-4 and C-5 have been removed in order to show the interior of the monument (Lozano Rodríguez et al.^25^). (**a**) Pillar P-3 with examples of biofacies (**a1**–**a3** observed in hand specimen). (**b**) Orthostat O-15 with examples of biofacies (**b1**–**b4** observed petrographically) and in hand specimen (**b5**). (**c**) Orthostat O-8 with examples observed petrographically (crossed polars) (**c1**,**c2**). (**d**) Orthostat O-5 with examples observed through the petrographic microscope (**d1**,**d2**). The star-shaped symbol indicates the place where a section was made for the petrographic study. Qtz: Quartz (designations after Kretz,^49^).

**Figure 2:**
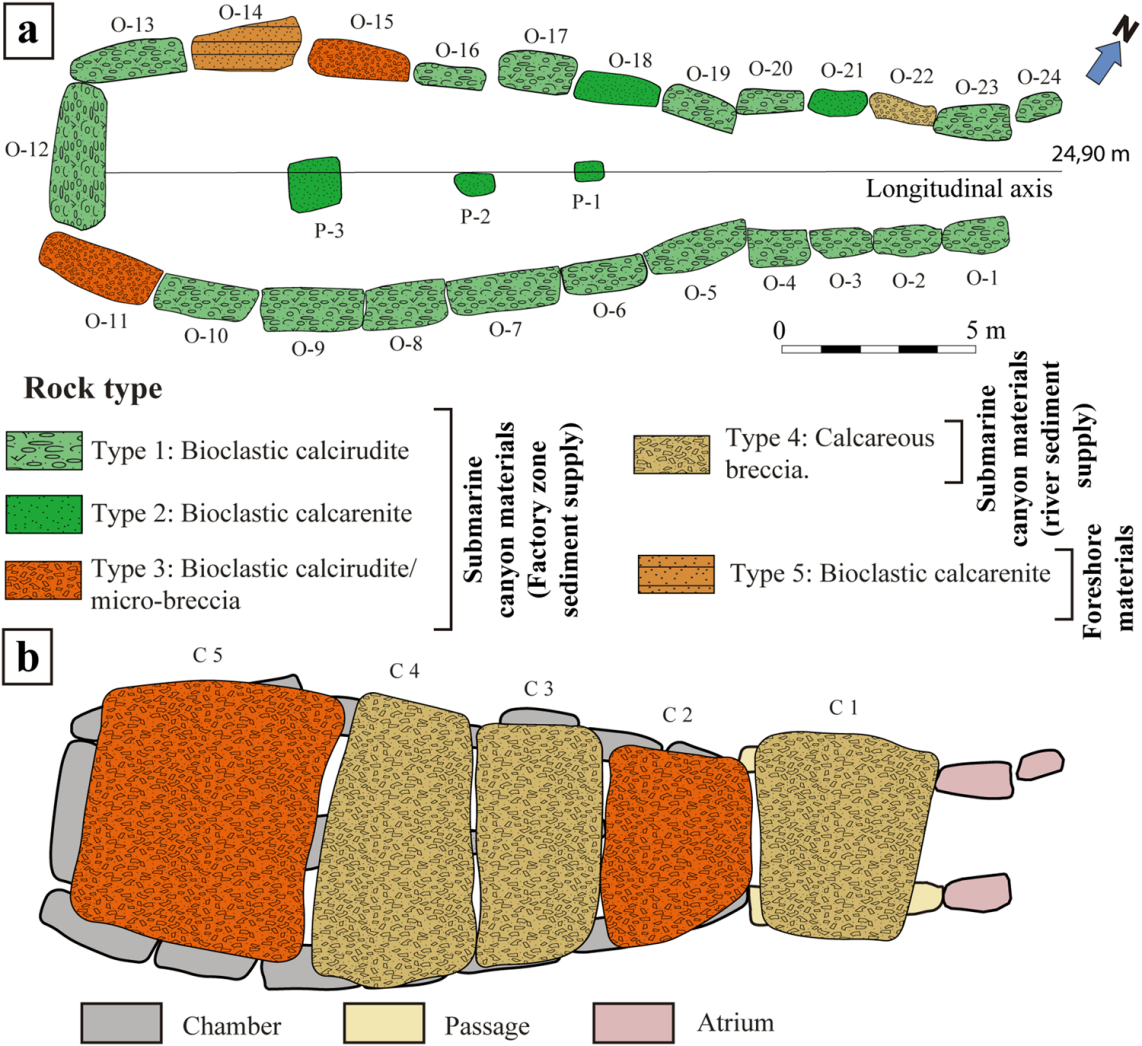
Schematic design of Menga showing the distribution of facies in the stones. (**a**) Without the capstones. (**b**) With the capstones (P: Pillar; C: Capstone; O: Orthostat) (Modified from Lozano Rodríguez, et al.^25^).

The 32 analysed stones (24 orthostats, 5 capstones and 3 pillars) belong to 5 different lithological types (Figs. 1, 2, Table 1). We have identified a first group of bioclastic sandstones in a broad sense and another one of breccias. Bioclastic sandstones commonly show a significant amount of subrounded quartz grains (sometimes in the form of a micro-breccia with clasts perforated by lithophagous organisms) poorly cemented by a shallow matrix of calcium carbonate (Fig. 1b–d). The breccias present a calcareous matrix and/or cement with sharp clasts of oolitic limestone, slate, ophites, marly limestone and iron oxides, with sizes ranging from medium sand to gravel.

**Table 1:** Types of stones comprising the mega-stones of Menga dolmen.

Type	Classification of limestones basad on the scheme of Folk^50^	Grain size (mm)	Mineralogical composition	Skeletal grains	Non-skeletal grains	Matrix	Texture classifications (Dunham^51^)	Microstructure and sedimentary structure	Sediment supply	Paleoenvironment
1	Bioclastic calcirudite	> 2	70–80% calcite, 25–18% quartz, limestone, iron oxides, feldspar and glauconite 5–2%	Main components: Bryozoans, bivalves (*Clamys*, pectinid) Other components: echinoids, coralline corals, benthic foraminifers (amphisteginas, globigerinas), brachiopods, balanids	Intraclasts (limestone) pellets	Low sparite and absence of micrite	Rudstone	Syndepositional intergranular voids, parallel-laminated	Factory zone	Submarine canyon
2	Bioclastic calcarenite	< 2	70–80% calcite, 25–18% quartz, limestone, iron oxides, feldspar and glauconite 5–2%	Main components: Coralline algae Other components: nodular and branching bryozoans, bivalves, solitary corals, echinoderm spines	Pellets	Contains carbonate mud	Packstone-rudstone, crusts are bindstones	Syndepositional intergranular voids, parallel-laminated	Factory zone	Submarine canyon
3	Bioclastic calcirudite/micro-breccia	> 2	70–80% calcite, 25–18% quartz, limestone, iron oxides, feldspar and glauconite 5–2%	Bivalves (pectinid), bryozoans, coralline algae	Pellets	Contains carbonate mud	Rudstone	Syndepositional intergranular voids, parallel-laminated	Factory zone	Submarine canyon
4	Calcareous breccia	> 2	70% calcite, 15% quartz, 8% feldspar, iron oxides (oncolites), sandstone and flint 2%. To a lesser extent: filositicates, slates, coal	Bivalves (pectinid), bryozoans, coralline algae	Dolomite, oolitic limestones, marly limestones	Contains carbonate mud	Rudstone	Synsedimentary cement, low-angle, parallel-laminated, overlapping edges	River	Submarine canyon
5	Bioclastical calcarenite	< 2	70–80% calcite, 25–18% quartz, iron oxides, feldspar and glauconite 5–2%	Bivalves (*Clamys*, pectinid), brachiopods, echinoderm spines bryozoans, echinoides	Pellets	Low sparite and absence of micrite	Rudstone	Syndepositional intergranular voids, low-angles, parallel-laminated, burrows		Foreshore (Beach)

Based on the classification of Dunhan^51^ for carbonate rocks and Folk^50^. Modified from Lozano Rodríguez et al.^25^.

Lithological Type 1 includes bioclastic calcirudite with rudstone textures and can be found in 18 out of the 24 orthostats. Type 2, which includes bioclastic calcarenite but with packstone-rudstone textures, is used for two orthostats (both on the right-hand side of the megalithic chamber at the monument's entrance), and in all three pillars. Type 3 corresponds to orthostats O-11 and O-15 and capstones C2 y C5, which were made with bioclastic calcirudite/micro-breccia with rudstone texture (Fig. 2). These three typologies are characterized by a mineralogical composition mainly of calcite and to a lesser extent quartz (Fig. 1b1,b3–4,c1–3,d1–2), limestone, iron oxides, feldspar and glauconite. The skeletal grains of these materials comprise fragments of bryozoans (Fig. 1a3,b2,c1,d1–2), bivalves (Fig. 1a2,b3,c1–2,d2), echinoderms (Fig. 1b4), coralline algae (Fig. 1a1,b1,d2), benthic foraminifers (Fig. 1c3,d1), and minor brachiopods and balanids with parallel lamination and syndepositional intergranular voids (Table 1). Type 4 corresponds to calcareous breccia with rudstone texture. This type appears in one of the stone orthostats (O-22) of the right-hand side of the chamber (at the entrance) and in capstones C1, C3 and C4. It is characterized by a mineralogical composition mainly of calcite, quartz, feldspar, iron oxides and flint, partly covered by sands. Skeletal grains are composed by fragments of bivalves, bryozoans, and coralline algae with syndepositional cement. Textures show low-angles, parallel laminated and overlapping edges (Table 1). Finally, Type 5 is found only in one of the stone orthostats (O-14) and is composed by bioclastic calcarenite with rudstone texture. Its mineralogical composition mainly consists of calcite and to a lesser extent quartz, iron oxides, feldspar and glauconite. This texture also shows skeletal grains composed of fragments of bivalves, brachiopods, spines from echinoderms, bryozoans, and echinoderms with syndepositional intergranular voids, low-angles, parallel laminated and burrows (Table 1).

In general, these stones range from soft to moderately soft according to the International Society for Rock Mechanics (ISRM) (Table 1). Our measurements show they are highly porous rocks with porosities ranging from 13.67% for type 1 (calcirudite), 13.29% for type 2 and 5 (calcarenite), 22.91% for type 3 (calcirudite/micro-breccia) and 29.62% for type 4 (breccia). We estimated apparent densities of 2321 kg/m^3^ for type 1; 2366 kg/m^3^ for type 2 and 5; 2237 kg/m^3^ for type 3 and 2318 kg/m^3^ for type 4 corresponding to real density of the stone materials of 2688 kg/m^3^; 2729 kg/m^3^; 2902 kg/m^3^ and 3294 kg/m^3^ respectively. Previous studies on the same rocks found similar apparent densities of 2264 for type 1; 2039 for type 2 and 2488 kg/m^3^ for type 4^30^ composed of poorly cemented, mostly bioclastic sandstones (see Fig. 1b–d). Simple compressive strength was 12, 80–34, 98 MPa for type 2, 15, 52–36, 15 MPa for type 1 and 22, 46–57, 30 MPa for type 4^30^. Type 2 from the same period in the nearby area of Granada, showed simple compressive strength of 13Mpa^30^ and apparent densities of 1741 kg/m^3^ ± 6^31^ and 1960± 0.39 kg/m^3^^32^, consistent with stones that can be easily carved.

### Characterization of the Upper Tortonian materials surrounding the Menga Dolmen

**Figure 3:**
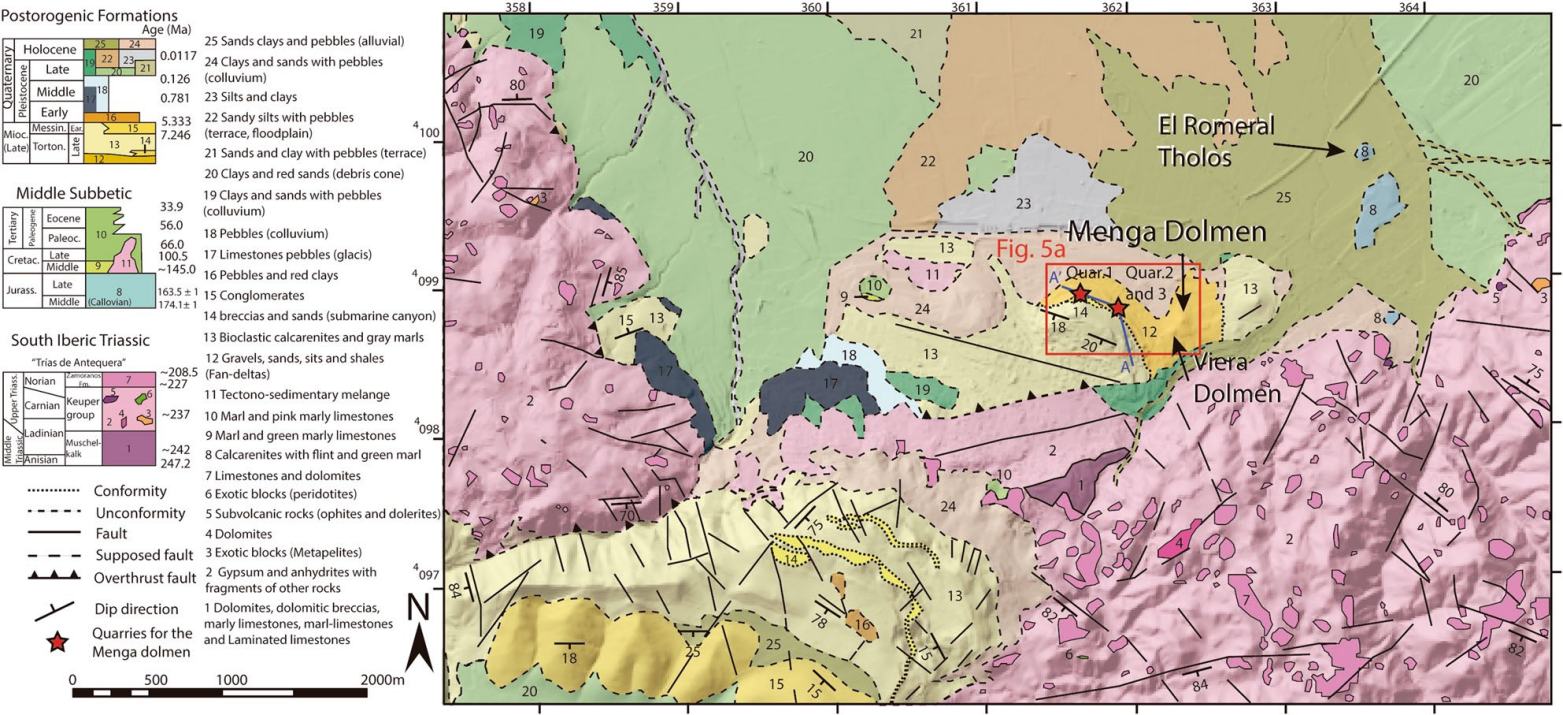
Detailed geological map on DTM of the area surrounding Menga, made with Adobe Illustrator based on new field data incorporated to pre-existing geological maps^47^ DTM data^48^. The blue line A-A' represents the direction of the stratigraphic diagram in Fig. 4a.

In the surrounding area of Menga, materials of Upper Tortonian age include rocks generated in a temperate carbonate palaeoenvironment. These carbonates occurred in a narrow platform observable in small outcrops through the northern margin of El Torcal, a massive karst formation located 11 km south of Antequera. These rocks are interspersed with other detrital sedimentary materials of the same age that rest on a substrate of Triassic (~252–~201 Ma) and Jurassic (~201–~145 Ma) materials (Fig. 3).

In the Antequera calcarenites, the factory paleoenvironment is virtually eroded -with the exception of a small outcrop at the southwest of Antequera city (Fig. S4), yet a sequence of a filled submarine canyon can be identified. This submarine canyon resulted from rivers that penetrated towards the marine platform, and whose head became totally individualized, without continental inputs. The submarine canyon was later filled with sandy and silty sediments carried by longshore currents. Over time, the infill of the submarine canyon was formed in several phases. The first phase was a marine regression with erosion. The second phase involved partial abandonment of the sedimentary load transported by the submarine river, generating conglomerate deposits at the beginning of the marine transgression. The last phase involved the upfilling of the submarine canyon with sediments, in the final stage of the transgression. A similar process has been described for the palaeo-bay of Alhama de Granada^33^.

At the base of the stratigraphic column there are uncemented sands, polygenic and heterometric (fine and coarse) gravels, larger boulders wrapped in a matrix rich in fine sediments and small layers of silts and shales. These uncemented materials, which contain abundant soft and angular clasts, extend over the area on which both dolmens were built (Fig. 3) and represent a high-energy environment. These are the facies that allow us to interpret the typical paleoenvironment of fan-delta materials.

**Figure 4:**
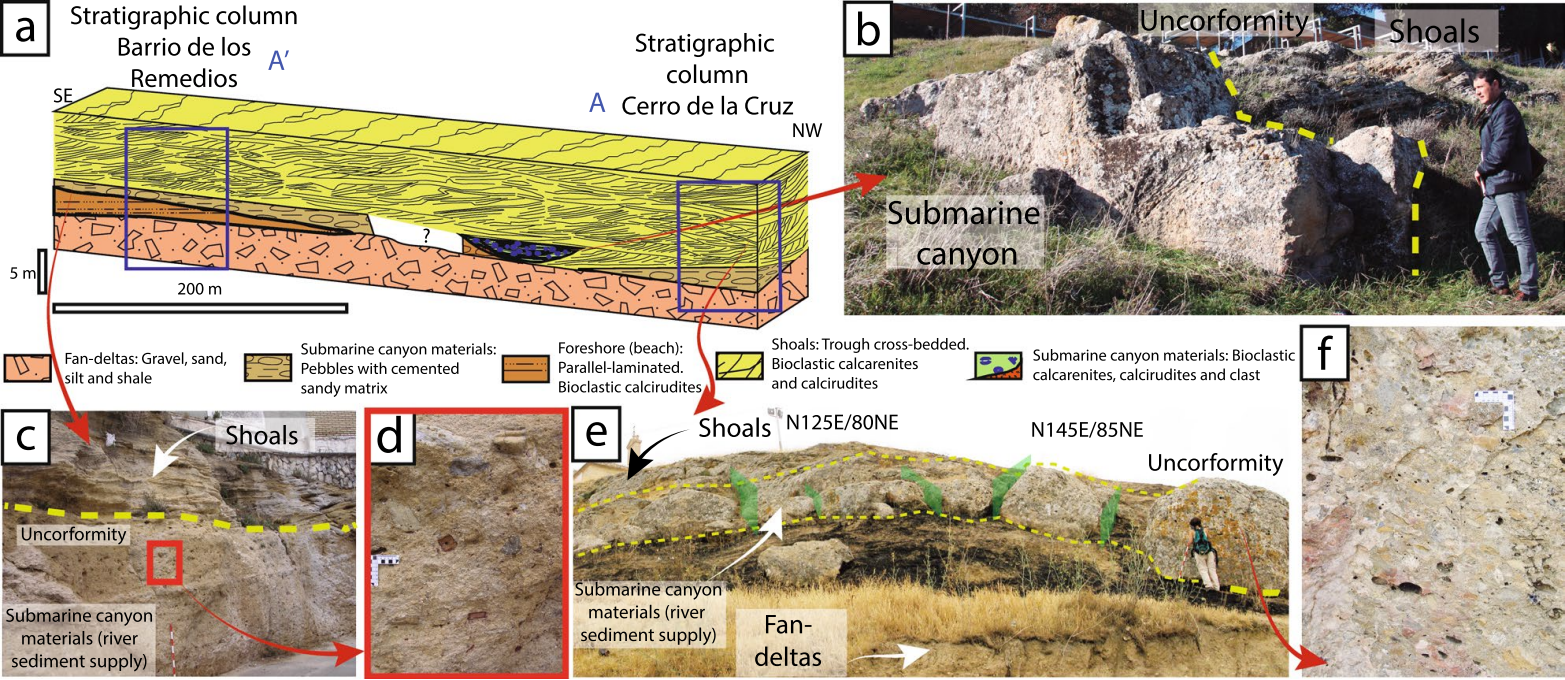
(**a**) Stratigraphic correlation of the Upper Tortonian sedimentary materials in the Los Remedios neighbourhood and Cerro de la Cruz. (**b**) View of the small outcrop of type 1 and 2 stones (submarine canyon), embedded by erosion in the shoals materials, preserved at Cerro de la Cruz. (**c**) Stratigraphic section at Los Remedios neighbourhood, showing the submarine canyon materials below the shoals. (**d**) Detail of the matrix-supported marine materials with large amounts of pisoliths and minor bryozoans and red algae or lamellibranchs. (**e**) Overview of the submarine canyon materials at Cerro de la Cruz, above the fan-deltas. Note the subvertical tectonic fracturing, which is perpendicular to the valley, displaying little tectonic penetration at metric scale, which favoured the extraction of large blocks. (**f**) Detail of well-cemented sedimentary materials at Los Remedios neighbourhood, with smaller amounts of pisoliths and local bryozoans and red algae or lamellibranchs.

**Figure 5:**
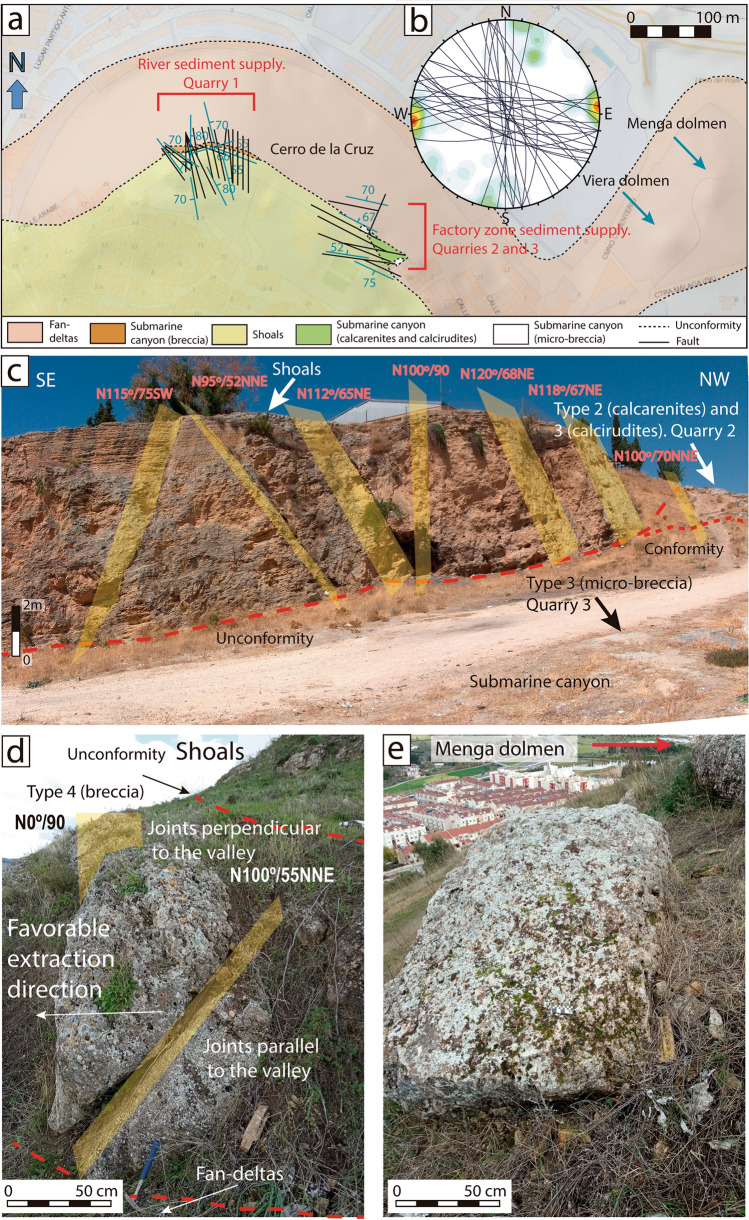
(**a**) Geological map of tectonic jointing on DTM, showing the location of Menga and Viera and the likely quarrying areas at Cerro de la Cruz. (**b**) Stereographic representation of the groups of joints. (**c**) Overview of the tectonic fracturing present in quarry areas #2 and #3. (**d**) Groups of joints observed in Quarry #1. (**e**) Example of a possible discarded megalithic stone at Quarry #1. Maps made with Adobe Illustrator based on new field data incorporated to pre-existing geological maps^47^ DTM data^48^.

Fan-deltas face towards the interior of today’s Antequera Depression. Overlaying those materials, with a thickness of about 4 m and in a discordant manner, lay bioclastic calcirudites very well classified with parallel lamination, corresponding to a foreshore (beach) paleoenvironments. These materials can only be seen in the stratigraphic column at Los Remedios neighbourhood, inside modern-day Antequera, about 700 m south-west from Menga (Fig. 4a). On these materials, and also in a discordant manner, there is a polygenetic and heterometric calcareous breccia, with high-energy oriented pebbles and some presence of perforations by lithophagous organisms and iron oxides. The clasts of this fabric are locally encumbered by poorly cemented, well classified and subrounded sands (Fig. 4c,d). Similar materials can also be seen in the stratigraphic column at Cerro de la Cruz (Fig. 4a,e,f) in stratigraphic correlation under the shoals’ palaeoenvironment and above the fan-delta one. However, in this location, the breccia is the best cemented and the amount of iron oxides is the lowest, corresponding to a submarine canyon palaeoenvironment (river sediment supply). Halfway between the stratigraphic columns of Los Remedios neighbourhood and Cerro de la Cruz, but closer to the latter, there is a small outcrop (currently only about 10 m^2^ are preserved) that includes bioclastic calcarenites and calcirudites, with large amounts of bryozoans, coralline red algae, bivalves and pebbles (Fig. 4b). This outcrop changes laterally towards a micro-breccia, extending a few meters farther (Fig. 5a). This lithology corresponds to submarine canyon paleoenvironments, in this case with a factory zone sediment supply, embedded in a discordant manner above the shoals in the NW (Figs. 4a,b, 5a and S4a–c). The shoals’ materials are the thickest ones, reaching up to 8 m at some points. They are bioclastic calcarenites and calcirudites with through cross-bedding (Figs. 4a–c,e, 5a and S4b).

### Connecting the dolmen and field lithologies

**Figure 6:**
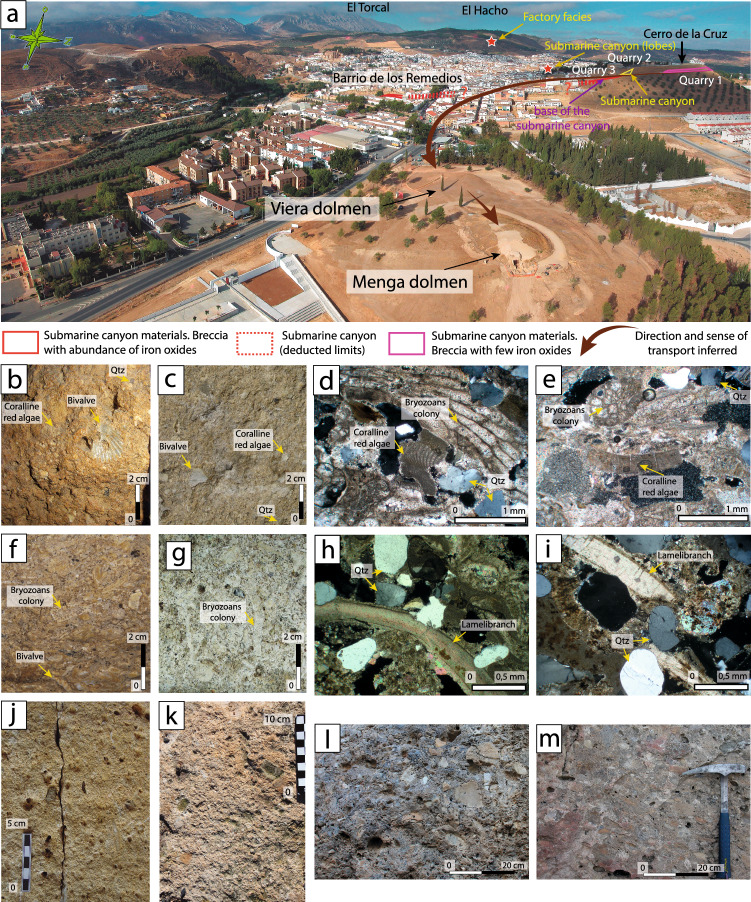
(**a**) Overview of Menga, Viera, Los Remedios neighbourhood and Cerro de la Cruz from the NE, showing the location of submarine canyons, the factory zone, lobes outcrops (red star) and possible quarrying areas. The direction of stone transportation from the quarries to Menga hill is suggested. (**b**,**c**) Detail naked eye of bioclastic calcirudite (lithology Type 1) of in Menga's orthostat O-10, and quarry #2 respectively. (**d**,**e**) Petrographic microphotography of orthostat O-5 (lithology Type 1), and quarry #2 respectively. (**f**,**g**) Detail naked eye of bioclastic calcarenite (lithology Type 2) of in Menga's pillar P-3, and quarry #2 respectively. (**h**,**i**) Petrographic microphotography of orthostat O-18 (lithology Type 2), and quarry #2 respectively. (**j**,**k**) Detail naked eye of bioclastic micro-breccia (lithology Type 3) of in Menga's capstone C-2, and quarry #3 respectively. (**i**,**m**) Detail naked eye calcareous breccia (lithology Type 4) in Menga’s capstone C-1, and quarry #1 respectively. All photomicrographs are crossed polars light.

The rocky outcrop of lithology Type 3 is designated as Quarry #3 (Figs. 2, 3, 5a,c, 6a) corresponding to orthostats O-11, O-15 and capstones C-2 and C-5 (Fig. 2) at the bottom of the submarine canal in which they formed. The stones of this outcrop are identical to those observed in the dolmen (Fig. 6j,k, Tables 1, 2) showing clasts of 0.5–2 cm in size, and small pebbles (1–2 cm), bioclastic, carbonate-matrix and pisoliths. The extensive exploitation of Quarry #3 area in historical periods has prevented us from finding more precise elements of Neolithic quarrying. In this quarry, fractures tend to be arranged verticality, with penetrative spacing of up to 7.5 m (Fig. 5a–c) compatible with the dimensions of capstone C-5 (6.95 × 6 × 1.88 m^30^).

**Table 2:** Lithological characteristics of the possible quarries that supplied the Menga dolmen.

Type	Classification of limestones based on the scheme of folk	Grain size (mm)	Mineralogical composition	Skeletal grains	No-skeletal grains	Matrix	Texture (Dunhan classification)	Microstructure and sedimentary structure	Sediment supply	Paleoenvironment	Quarry
1	Bioclastic calcirudite	2–25	70–80% calcite, 25–18% quartz, limestone, iron oxides, feldspar 5–2%	Main components: Bivalves (Clamys, pectinid), bryozoans Other components: Coralline corals, echinoids, benthic foraminifers (amphisteginas, globigerinas), brachiopods	Intraclasts (limestone, pellets)	Low sparite and absence of micrite	Rudstone	Syndepositional intergranular voids, parallel-laminatid	Factory zone	Submarine canyon	2
2	Bioclastic calcarenite	< 2	70–80% calcite, 25–18% quartz, limestone, iron oxides, feldspar and glauconite 5–2%	Main components: Coralline algae Other components: Bivalves, branching bryozoans, echinoderm spines	Pellets	Contains carbonate mud	Packstone-rudstone, crusts are bindstones	Syndepositional intergranular voids, parallel-laminatid	Factory zone	Submarine canyon	2
3	Bioclastic calcirudite/micro-breccia	> 2	70–80% calcite, 25–18% quartz, limestone, iron oxides, feldspar and glauconite 5–2%	Bivalves (pectinid), coralline algae, briyozoans	Pellets	Contains carbonate mud	Rudstone	Syndepositional intergranular voids, parallel-laminatid	Factory zone	Submarine canyon	3
4	Calcareous breccia	> 2	70% calcite, 15% quartz, 10% feldspar, iron oxides, (oncolites), sandstone and flint 2%. To a lesser extent: coal, slates	Bivalves, coraline algae, bryozoans	Oolitic limestone, dolomite, marly limestones, ophites	Contains carbonate mud	Rudstone	Synsedimentary cement, overlapping edges, parallet laminated, low-angle	River	Submarine canyon	1

Based on the classification Dunhan^51^ for carbonate rocks and Folk^50^.

In the yard of the La Trinidad Church, located 1.6 km westward of the dolmen, there are outcrops of submarine canyon materials (Fig. S4a–c). However, these are lobe type, and therefore different from those observed in the great dolmen. Also, these rocks show a highly penetrative fracture setting of metric scale, which would render impossible the extraction of large stone blocks. All three candidate quarrying areas stand on soft earthen delta materials, making them ideal quarrying locations.

The outcrop of lithology Type 4 is clearly observable at Cerro de la Cruz, in Quarry #1, ca. 850 m westward of Menga (Figs. 3, 4a,e,f and 6a,m). These materials can also be found at Los Remedios neighbourhood. However, there are clear differences between the two outcrops. Matrix cementation is greater and the amount of pisolith is smaller in Cerro de la Cruz Quarry #1 than in Los Remedios neighbourhood, which is consistent with the lithology of some of the stones in the dolmen (O-22, C-1, C-3 and C-4). Therefore, their origin must be Quarry #1 at Cerro de la Cruz displaying the same type 4 materials (micro-breccia) (Fig. 6a,l,m, Tables 1, 2).

The outcrop of lithology Type 5 presents a very characteristic foreshore materials parallel-laminated formation observed in orthostat O-14. The parallel-laminated formation has caused buckling to orthostat O-14 likely due to the mound and the vertical stress generated by capstone C-5, as these planar discontinuities are unsuitable for this type of monument^34^ (Fig. S3e). Although this problem has only been noted for this stone, it is, undoubtedly, a design’s flaw of this part of the dolmen. We do not know the origin of this Type 5 lithology, due to the poor conservation conditions of the quarries.

The petrographic study also reveals that the grains of the five lithological types described for Menga are poorly cemented (Figs. 1b1–4,c1–3,d1–2, 6d,h).

This area has been affected by the Alpine tectonic movement, with widespread fracturing, mostly subvertical, which can be seen clearly in the most likely quarrying areas (Figs. 4e, 5a–d). These fractures generated various groups of joints (Fig. 5). In the area designated as Quarry #1, the fracturing presents a prevailing N–S direction, subvertical, or sloping to the E, but others slope to the NNE and, to a lesser degree, to the WSW, and there is even some not-too-vertical ESE fracturing sloping towards the NNE (Fig. 5c). In some cases, these fractures intercept each other. In the areas designated as Quarries #2 and #3, the fracturing is predominantly NE-SW with some NNE, and to a lesser extent SSW, incline (Fig. 5).

The tectonic fracturing present at Cerro de la Cruz is subvertical with barely penetrative spacing at metric scale, thus generating isolated blocks of sizes compatible with those of the orthostats, capstones and pillars of Menga. There is a group of fractures presenting inclination planes of 55° towards the valley and the Menga hill, which was obviously a favourable element for their extraction in Neolithic times (Fig. 5d).

The materials observed at Cerro de la Cruz fully match those identified at Menga. Their topographic position, ca. 100 m above the Menga hill, favoured downhill transportation of the stones. For both reasons, Cerro de la Cruz is the most likely quarrying area for the construction of the dolmen (Fig. 6a). In fact, a non-systematic archaeological survey of this area carried out while analysing the geology led to the recovery of some hand-thrown fragments of pottery compatible with a Neolithic manufacture.

## Discussion

Menga is located in one of the most complex geological areas of the Iberian Peninsula, rich in abiotic resources (Supplementary Text S2). The ophites, dolerites and flint quarries in the Antequera surroundings played a major part in the process leading up to the construction of the great dolmen, as most of the tools (hammers, maces, axe heads) used in the quarrying and dressing of the stones were made in those materials (e.g.,^35–37^).

The hilltop where Menga was built had also a relevant history of human activities^24,35^, and a high social and ritual significance by the time the dolmen was erected. Apart from its earlier history, this hill was selected to accommodate the great dolmen mainly based on three major locational advantages: (i) it was the only position from which its chamber would be aligned with both La Peña de los Enamorados, the anthropomorphic limestone massif presiding over the easterly horizon, and summer, which produces a complex pattern of light and shadow inside the chamber (average of 22°); (ii) the proximity to the quarry used for its construction as revealed by our results; i.e. the topographic position of those outcrops on a higher elevation (namely Cerro de la Cruz), allowed for a downhill transportation of the massive stones; (iii) Menga designers avoided soft soils enriched in marls and clays in the surrounding areas, as they would not grant the stability of the monument on the ground, and, thus, placed it on the much more stable breccia and sand lithologies (fan-delt) of the hilltop.

**Figure 7:**
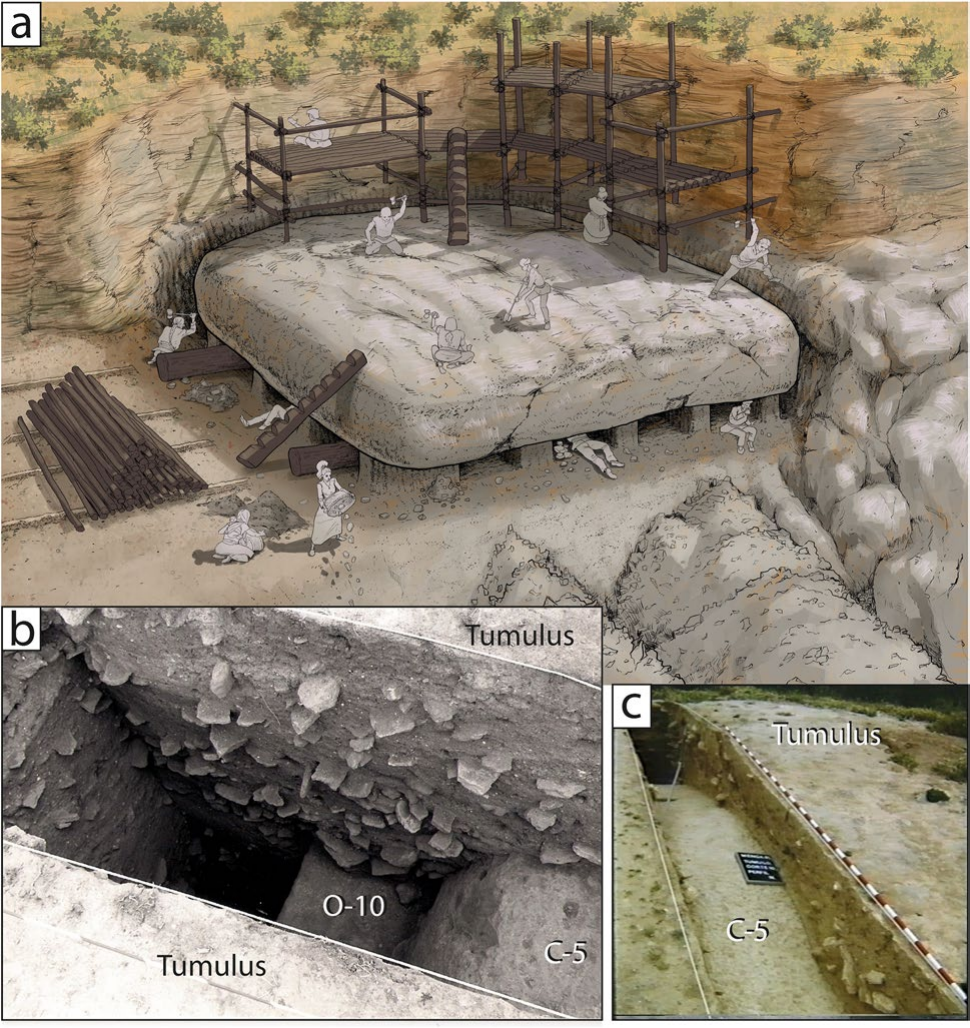
(**a**) Artistic representation of quarrying activities for the extraction the capstone C-5 in Cerro de la Cruz Quarry #2. Drawing: Moisés Bellilty under guidance of José Antonio Lozano Rodríguez and Leonardo García Sanjuán. (**b**) Aspect of the thickness and shape of the C-5 capstone, the support on part of the O-10 orthostat and the tumular structure. University of Malaga excavation. Ferrer-Marqués, 1984. Conjunto Arqueológico Dólmenes de Antequera. (**c**) Convex morphology of the top of the C-5 capstone and the thickness of the tumular structure. University of Malaga excavation. Ferrer-Marqués, 1984. Conjunto Arqueológico Dólmenes de Antequera.

The identification of these quarries has been possible through the petrological comparison of the different sedimentary facies of the stones used to build the dolmen with the surrounding geological outcrops (Supplementary Text S3, Tables 1, 2). Additional evidence about the quarrying area came from a detailed geological cartography, stratigraphic correlations, the thickness of the outcrops and an exhaustive study of tectonic fracturing in Cerro de la Cruz (proposed area for quarrying). It has been determined that tectonic fracturing produced stones large enough to be part of the dolmen (Fig. 5c,e). For the manufacturing of capstones, even larger stones were utilised, which were extracted from the different quarries through a very elaborate and well-planned process. Our hypothesis is that the stones were individualized at the base by means of small pillars as documented in ancient civilizations (e.g.,^38^) and well into historical times^39^ (Fig. 7a). We also hypothesize that the location of the dolmen was chosen as the result of careful planning and design of engineering works.

Remarkably, none of the other megaliths in the Antequera complex, even later ones, used stones as large as those used in Menga. In other southern Iberian dolmens such as Soto or Alberite large stones were used, but never reaching the dimensions of Menga’s. Mostly harder stones such as greywackes, sandstones or volcanic stones in the case of Soto^40^; and nodular, tabled and marble limestone in the case of Alberite^41^. In Iberia, quarries are usually located in the vicinity of the megaliths (e.g.,^42^). This is the case with Alberite (5 km away^41^) and Puigseslloses (~ 4 km^15^). In our case, Menga lies only about 850 m away from the quarries.

These soft stones are not very resistant to transportation, which must have been an additional complication in the construction process. Working with these large and fragile stones must have involved a massive labour investment not only in stone working, but also in wood-working and rope-making^43^. Large amounts of wood must have been used to build the scaffolding used in the quarrying process and to prepare the roads on which the massive stones were transported. Figure 7a presents an artistic reconstruction of the quarrying work of capstone C-5. In 1991, the University of Malaga carried out several archaeological excavations in the Menga dolmen. One of these excavations was in the area of the mound occupied by Capstone 5. Photographs taken at the time of the excavation have allowed us to know the morphology of Capstone 5 in its entirety (Fig. 7b,c). Menga’s Capstone 5, with an estimated weight of around of 149.59 ± 9.17 tons, is the second largest ever used as part of the megalithic phenomenon in Europe after the Grand Menhir Brisé at Locmariaquer (France), which has an estimated weight of 335 tons^42^ (Supplementary Text S4), and the larges stone ever used in a Neolithic dolmen (Figs. 2b, 7 and S5).

Among the four samples studied (corresponding to type 1–4 materials existing in the quarries, as for type 5 material we have not found its quarry), type 1 and 2 with similar densities typical of carbonate rocks, show the lowest porosity (around 13%). The type 3 and 4 samples are characterized by a higher porosity, reaching almost 30% in type 4. However, type 4 was made of denser materials with a high content in clasts of iron oxide.

This type of poorly cemented sandstone continues to be used today in southern Iberia. It can be found in other megaliths like Pantano de Los Bermejales^44^, and in many historic buildings^32,45^. However, the gaps between grains and the little cement in this porous rock can be occupied by water evolving through the rock by capillarity. This generates a significant problem for the stability of the building by increasing its weight and the risk of fragmentation^46^.

The builders of Menga solved these problems by isolating the largest stones with a tumulus designed to insulate it, keeping out rain water. This tumulus was made of alternating layers of carefully interlocking flat sandstones and pressed ground (Fig. 7b,c). The possible humidity that could penetrate the monument through the C-1 directly in contact with the elements was fixed by using a lithology that is not very porous and somewhat more cemented, such as the calcareous breccia.

## Conclusions

The full geological survey of the rocky outcrops found in an area of ca. 3 km radius around Menga indicates that Cerro de la Cruz was the most likely source of the stone materials used to build the great dolmen. Detailed sedimentological description made it possible to identify five different types of stone: (1) bioclastic calcirudite, (2) bioclastic calcarenite, (3) bioclastic calcirudite/micro-breccia, (4) calcareous breccia, and (5) bioclastic calcarenite (foreshores materials). These are mainly sedimentary soft rocks associated with platform and submarine canyon paleoenvironments.

The quarries located at Cerro de la Cruz, is identified from the sedimentological and fracturing perspectives. We propose that the stones of Mega were transported continuously downhill, across a gentle slope averaging 22° for a distance of approximately of 1 km. The nearby location and the natural fractures present at the quarries would have facilitated the extraction and transportation of the massive stones.

We conclude that the location of the quarries and geological features was an additional critical factor for the emplacement of Menga. The use of soft stones such as calcarenites allowed Late Neolithic communities to work gigantic stones. Neolithic communities display a deep knowledge of the geotechnical and geological properties of the stones used and the quality of the terrain chosen as foundation. They avoided marls, clays and unconsolidated lithologies for stone movement and monument emplacement. They carefully selected the substrate, used pillars and avoided water infiltration, among others, in order to prevent deterioration of these soft stones and ensure the stability of the dolmen. For this purpose, a waterproof tumulus was created.

The quarrying and transportation of the massive stones from Cerro de la Cruz to the hill of Menga must have demanded intensive planning, highly accurate logistics and enormous labour investments. From these results, it can be inferred that the woodwork associated with the construction process must have also demanded the use of large amounts of timber. Considering the ramp construction and the size, number (over 30 large stones) and fragility of the stones, the construction of Menga embodies a unique accomplishment representing the state-of-the-art in megalithic engineering in prehistoric Iberia and possibly in Europe. Menga stone C-5 emphasizes the magnitude of this achievement as it is the largest stone used in a composite megalithic monument and it is also a soft stone used as a cover slab which requires a high level of technical proficiency.

## Methods

Because of conservation reasons samples for thin-section analysis oof some of the Menga stones could not be taken. Hence, the study of the capstones was based on optical observations through a 10X and 4X monocular hand-lens. This method was also used in other elements of the dolmen, in which the fossils were present (Fig 1a). In addition, we complemented the petrographic study of the orthostats and pillars with both a microscopic reanalysis of thin-sections (Fig. 1b–d) from an earlier study^25^, and with new samples obtained from natural outcrops located in the surrounding area. The calculation of the mineralogy and the proportions of the main grain types was carried out quantitatively by means of an Olympus BHT petrographic microscope. Thus, this paper presents a full revision of the mineralogy, textures, palaeontology and sedimentary structures of the different sedimentary facies involved in the architecture of Menga, integrated with a new, more precise geological mapping of its environment.

The new geological cartography was based on pre-existing geological maps Spanish Geological Survey Institute (IGME in its Spanish acronym), specifically sheets #1023 and #1024) (IGME 1982, 1:50,000^47^), which were completed with extensive field work and data processing using Adobe Illustrator software and digital terrain models (DTM). DTM data were obtained from the National Geographic Information Centre of Spain (IGN^48^). A new detailed map 1:25,000 covering an area of 5 × 7.5 km around Menga was made allowing precise location of the abiotic resources and potential quarrying locations (Fig. 3). Stratigraphic sections were drafted where the quality of the surfacing materials was good enough, and correlations were established between them using tape measurements. Finally, the structural features (e.g. directions and dips) were measured using a Freiberger compass.

Special attention was paid to tectonic fractures and their orientation in potential quarrying areas, in order to compare the known size of the stones used in the dolmen with the natural spacing of the tectonic fractures. The orientation of the fractures was determined through stereoscopic diagrams (see also^20^) by using Dips software 7.0 (Rocscience).

Each lithological group was determined as a function of its grain size, mineralogical composition, skeletal and detrital grains, matrix, microstructures and sedimentary structure. The labelling of the Menga stones involved in the study reflects earlier work^37^ and was made according to their position in the dolmen: (i) O (Orthostat), slab or large block of stone placed vertically; (ii) P (Pillar), resistant structural element with support function; and (iii) C (Capstone), roofing stones that rest on the previous ones (Fig. 2a).

The apparent density and open porosity of source rock samples were determined by means of mercury intrusion porosimetry (MIP) using a Micromeritics Autopore V 9600 porosimeter reaching a maximum pressure of 227 MPa. These analyses were carried out in the Department of Mineralogy and Petrology of the University of Granada. One sample of each stone type was analysed.

### Supplementary Information

Supplementary Information.

## Data Availability

DIP directions, porosity and density data are available from the CSIC data repository http://hdl.handle.net/10261/304960. Any other information used and/or analysed during the current study are available from the corresponding author on reasonable request.
