# Supplementary material for: The provenance of the stones in the Menga dolmen reveals one of the greatest engineering feats of the Neolithic
Source: Sci Rep. 2023 Dec 1;13:21184. doi: 10.1038/s41598-023-47423-y (PMC10692229; doi:10.1038/s41598-023-47423-y)
Supplement: Supplementary file 1 — Supplementary Information. [file 41598_2023_47423_MOESM1_ESM.docx]

**Supplementary Information**

The provenance of the soft stones in the Menga dolmen reveals one of the largest engineering feats of the Neolithic

José Antonio Lozano Rodríguez, Leonardo García Sanjuán, Antonio M. Álvarez-Valero, Francisco Jiménez-Espejo, Jesús María Arrieta, Eugenio Fraile-Nuez, Raquel Montero Artús, Giuseppe Cultrone, Fernando Alonso Muñoz-Carballeda, Francisco Martínez-Sevilla

Corresponding author: José Antonio Lozano Rodríguez; ja.lozano@ieo.csic.es

**This supplement comprises**

Supplementary Figures S1‐S6

Supplementary Texts S1‐S4

References for Supplementary Texts

**Supplementary Figure S1**


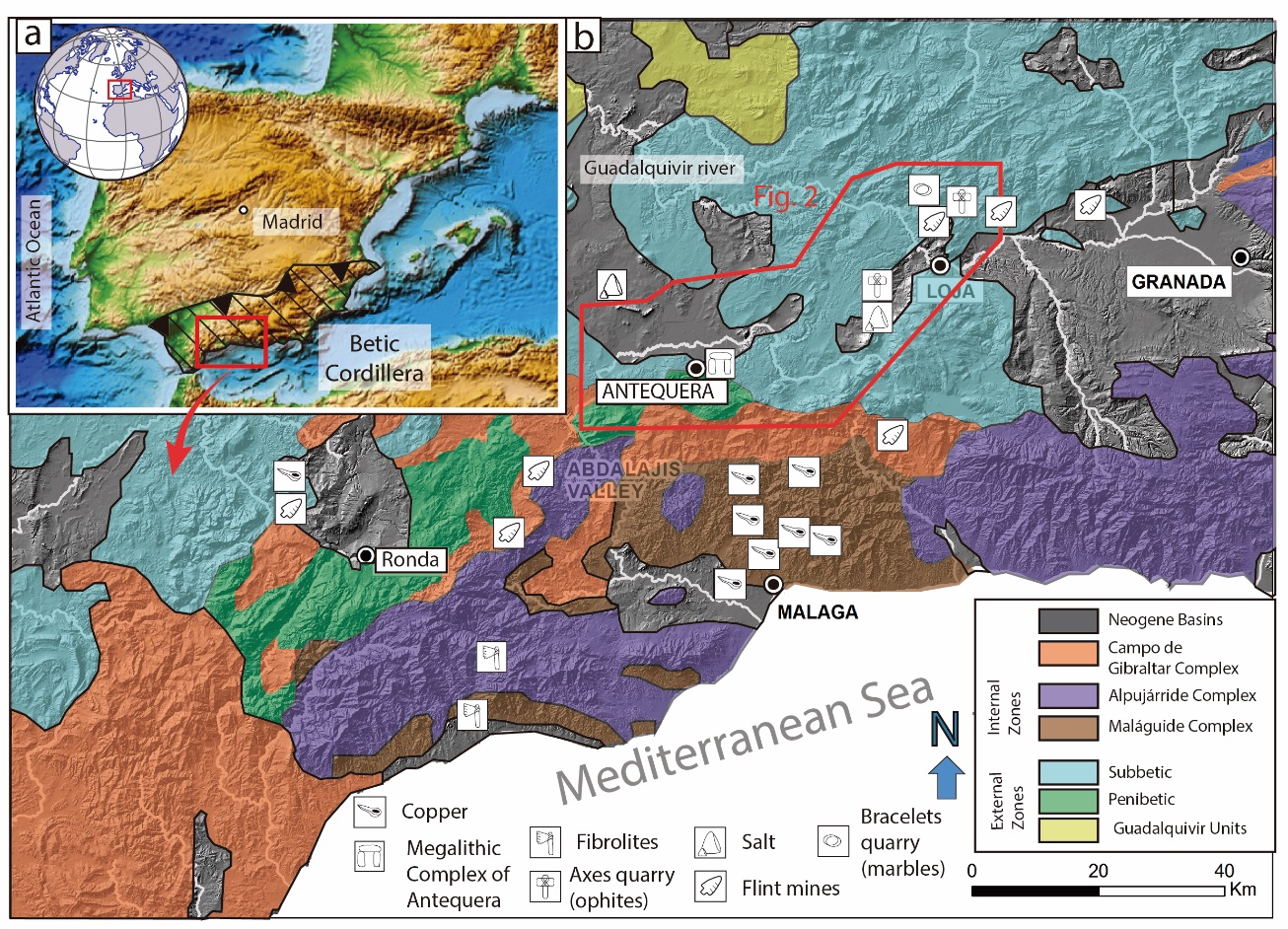


**Figure S1.** (a) Location of Antequera in the Iberian Peninsula, showing the Betic Cordillera (modified from Lozano Rodríguez et al.^1^. (b) Geological map on DTM of the central-western Betic Cordillera, showing the location of the Antequera megalithic site and abiotic resources locally available. Source of the DTM data: National Geographic Information Center of Spain (IGN)^2^.

**Supplementary Figure S2**

**
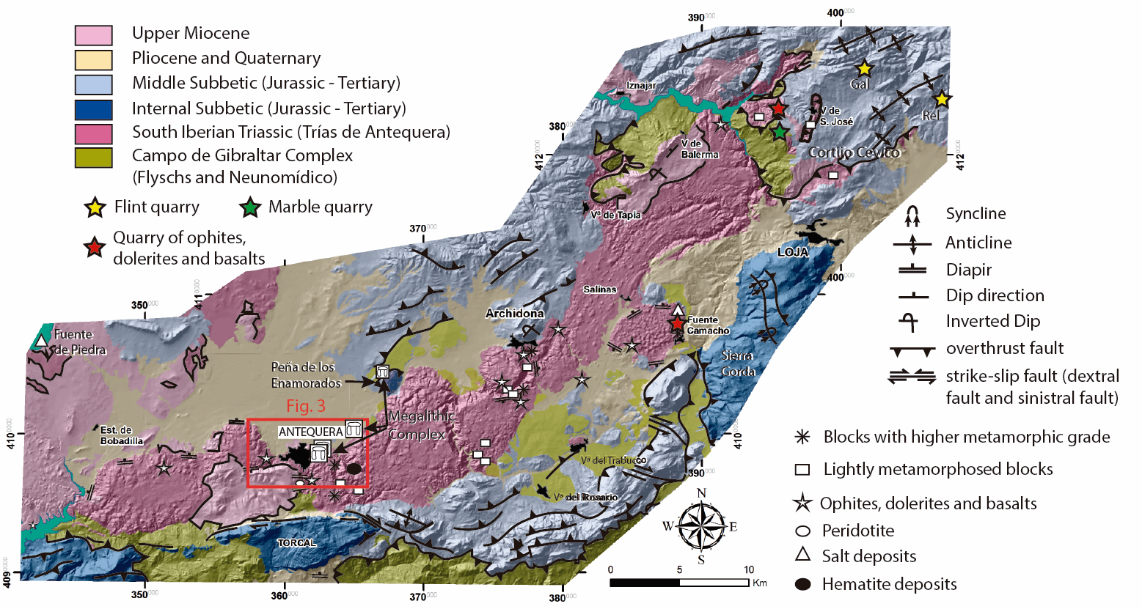
**

**Figure S2.** Geological map on DTM of the “Trías de Antequera” and surrounding materials with related abiotic resources. Source of the DTM data: National Geographic Information Center of Spain (IGN)^2^.

**Supplementary Figure S3**

**
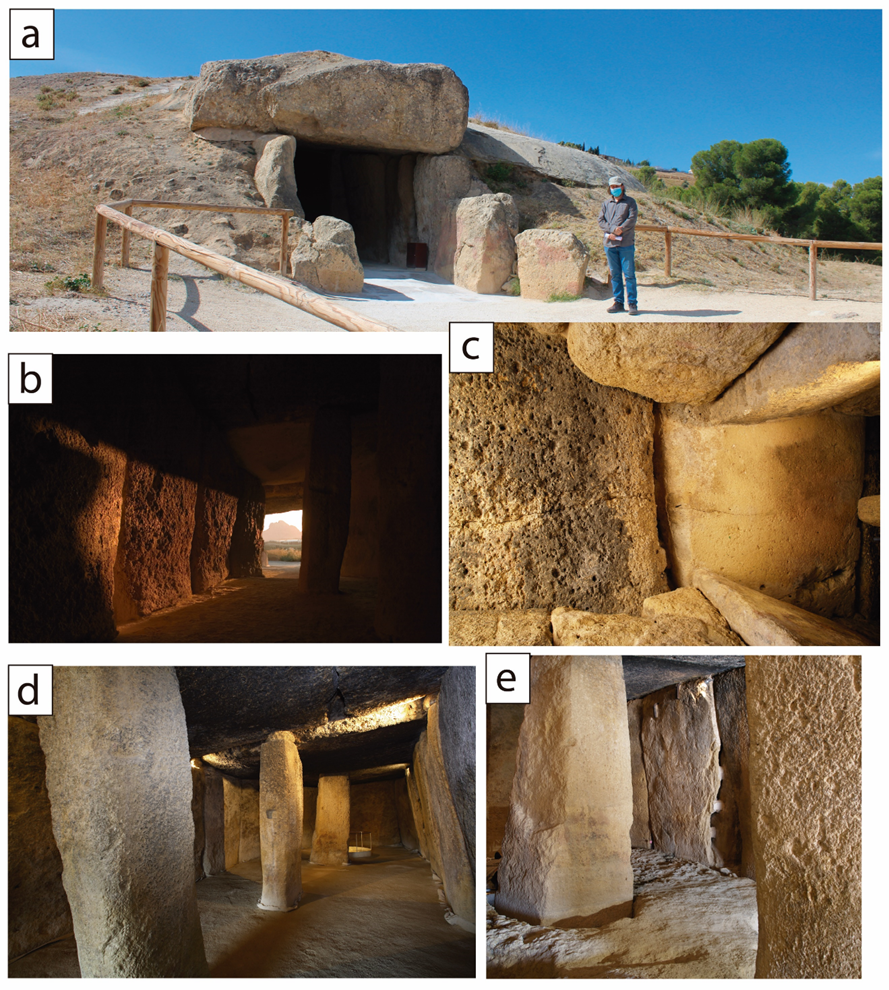
**

**Figure S3.** (a) Entrance to Menga. (b) View of La Peña de los Enamorados from Menga. (c) Capstones (C-1 on the left and C-2 on the right). (d) Interior of Menga, and three pillars currently preserved. (e) Interior of Menga with pillars (P-3 on the left and P-2 on the right); upright O-14, of foreshore facies (beach), can be seen between both pillars.

**Supplementary Figure S4**


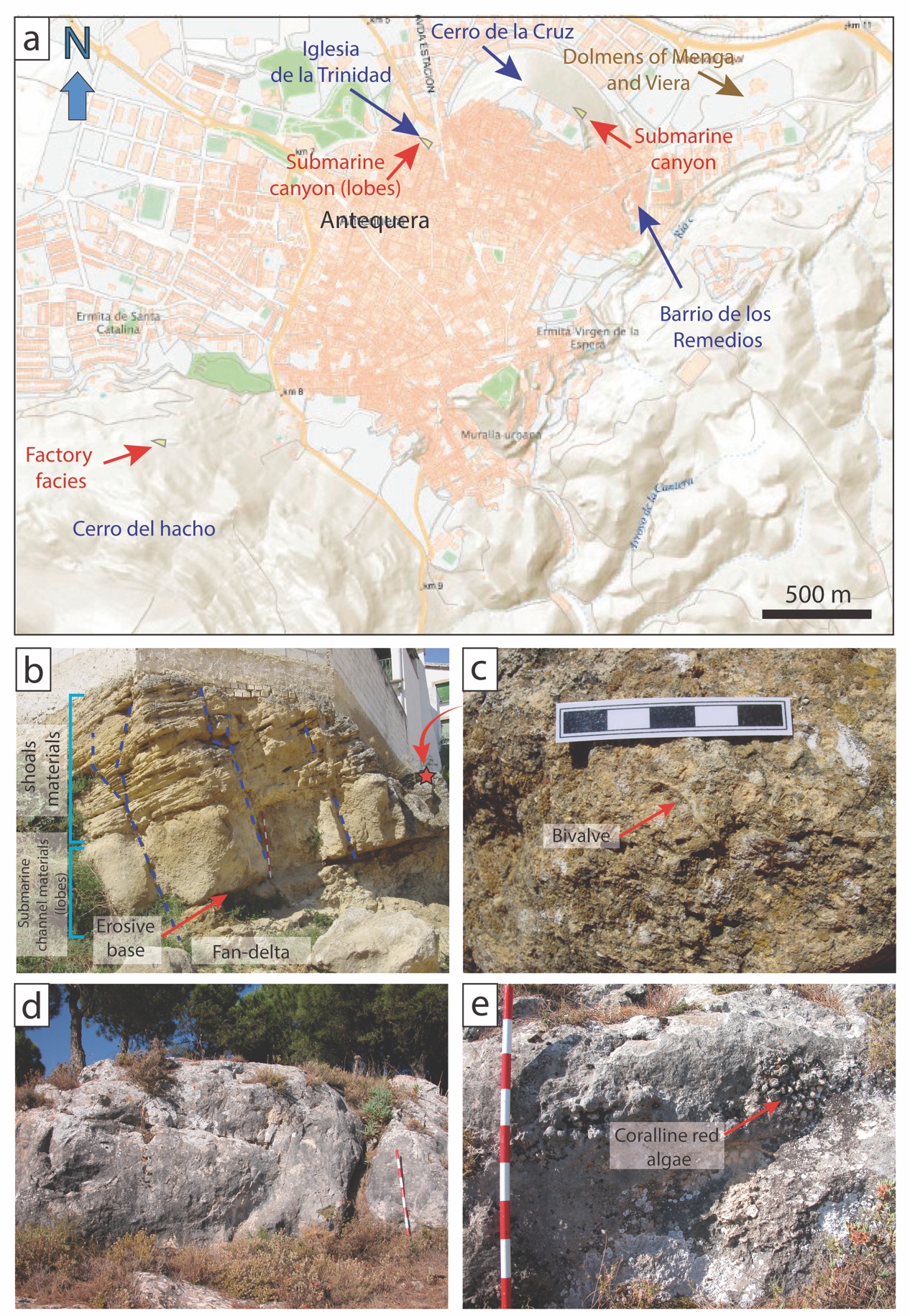


**Figure S4.**  (a) Topographic map of the study area with the outcrops locations of the factory facies, submarine canyon and lobes. (b) Overview of the lobes and tectonic fracturing penetrative at metric scale. (c) Detail naked eye of the microfacies with lobes bivalves. (d) View of the factory facies embedded between the shoals materials (above) and the fan-delta (below) at the back of La Trinidad church, in Antequera (note the penetrative tectonic fracturing at metric scale). (e) Detail of the coralline red algae facies occurring between the factory facies.

**Supplementary Figure S5**

**Figure S5.**  Schematic design of the capstone (C 5) in plan^3^.

**Supplementary Figure S5**

**Figure S6.**  Schematic cross-section of the capstone (C 5) perpendicular to the longitudinal axis of the dolmen.

**List of Supplementary Texts**

Text S1. Geographical and geological context.

Text S2. Chrono-cultural framework and key position of Menga Dolmen.

Text S3. Lithological characterization of other similar materials from the Upper Tortonian.

Text S4. Calculation of the approximate weight of capstone C 5.

**Text S1. Geographical and geological context**

The Antequera megalithic site comprises four megaliths: the Menga and Viera dolmens, the El Romeral tholos and the newly discovered La Peña megalithic tomb^4^. Geographically, the site is located at 500 m above sea level on the southern edge of the Antequera basin (37° 1’31.62” N, 4° 32’48.97” W)^1^. Antequera enjoys a favourable geographic and strategic location, connecting the interior of the Betic Cordillera with the Guadalquivir valley, the Mediterranean seaboard and the Iberian mainland. Basically, Antequera hinges between the Atlantic and the Mediterranean, acting as a crossroads (Fig. S1).

Geologically the site stands in the External Betic Zones (Fig. S1a), located to the south and south-east of the Iberian Massif and Guadalquivir Valley and formed by sedimentary materials deposited during the Mesozoic and, partly, the Cenozoic (e.g.,^5–8^). Specifically, the Subbetic Zone is located in a distal paleogeographic position in relation to the foreland (e.g.,^5,6^), composed of pelagic and hemipelagic rocks, with ages ranging from the Triassic to the Lower Miocene (e.g.,^9^). The Guadalquivir Units appear on the limit of the Cordillera itself and the Guadalquivir Basin, forming a set of allochthonous and chaotic units^10^. For its part, the South Iberian Triassic constitutes the oldest Germanic facies materials deposited in the South Iberian Paleomargin (Fig. S1b).

The entire southern and north-western edge of the Antequera region comprises materials from the "Trías de Antequera" paleodomain (e.g.,^11,12^). Ultimately, this represents a South-Iberian Triassic resting under part of the Jurassic age materials of the Subbetic (Fig. S2), as a tectonic mélange with blocks, some of hectometric sizes and larger, many of them exotic from Triassic to Tertiary age with a degree of metamorphism in some of these blocks^11^. The calcareous materials of the Internal Subbetic outcrop, such as La Peña de los Enamorados, as well as the Middle Subbetic are found to the northeast of the Antequera area (Fig. S2).

In the Antequera area, detrital deposits typical of marine temperate carbonates are abundant, with a predominance of bioclastic calcarenites and calcirudites and minor breccias and conglomerates. These detrital materials result from the paleo-relief existing around this paleo-sea in the Late Miocene, which provided sandy silt and marls for its innermost zones. Calcarenites were deposited in a shallow marine platform crossed by submarine canyons filled by calcarenites, calcirudites and breccias, as described in other locations in South Iberia^13^. These materials date to the Upper Tortonian, containing Blobigerina decoraperta, Glogigerinoides obliqus, Turborotalia humerosa, etc,^14^. At the top of the marine sequence, we found Messinian (7,246-5,333 Ma) conglomerates with discordant position. During the Quaternary, the Guadalhorce river generated accumulations of alluvial material forming fluvial terraces^1^.

**Text S2. Chrono-cultural framework and key position of Menga Dolmen**

By the end of the 5th millennium BCE, a major shift occurred in the development of monumentality across southern Iberia (e.g.,^15–19^). Two types of monuments that would preside over the social life of Iberian peoples in the following two millennia became widespread: ditched enclosures^19,20^) and megalithic chambers (e.g.,^15,17^). Both phenomena were closely connected, and occurred in parallel with major underlying cultural processes, such as the appearance of larger permanent or semi-permanent open-air settlements, demographic growth, intensification of agropastoral production and a gradual increase in social complexity^21^. The long cycle of ritual monumentality that started at the end of the 5th millennium BCE was to yield some truly remarkable creations, both in terms of their scale and architectural design, as well as in the social practices they were part of.

At Antequera ritual monumentality materialized impressively. The region had favourable geographical and ecological conditions for it, including a key position within the southern reaches of Iberia, a unique year-round plentiful supply of fresh water, and a wealth of both biotic and abiotic resources. The locally available lithic resources included flint, hard rocks for manufacturing macro-lithic tools, such as basalts, ophites and dolerites, as well as other much sought-after materials like ochre, copper and salt (Figs. S1, 2). Regarding flint, one of the most important rock for late prehistoric societies, there are several outcrops in the surrounding regions, including the “Milanos formation” (e.g.,^22,23^), the “Turón Valley” outcrop^24–26^, and the “Malaver formation” in the Ronda Basin. Quarrying activities dating to the Neolithic and Copper Age have been identified in all three of them. Hard rocks, such as ophites, dolerite and basalt, are present at the called "Trías de Antequera” geological formation. It is worth noting the proximity of copper carbonates from the “Montes de Málaga” area, which were used for copper smelting activities^27^ (Fig. S1). Another crucial abiotic resource for livestock and food preservation was salt. In the “Trías de Antequera” there are several locations in which salt occurs naturally, in connection with evaporite diapirs. In at least two of this diapiric outcrops, evidence of prehistoric exploitation has been observed: Fuente de Piedra and Fuente Camacho (Fig. S1, 2) (e.g.,^11,28^). In addition, the “Trías de Antequera” provides marble sources used to manufacture bracelets during in the Early Neolithic^29,30^, and pigments like ochre associated with multiple activities such as ceramic and bodily decoration, rock art, burials, or hide-working^31^.

The earliest proto-megalithic construction in Antequera is located at Arroyo Saladillo (4000 to 3900 BCE), a site located 6 km west of Menga. Arroyo Saladillo is also among the earliest megalithic burials in the Iberian Peninsula^32,33^.

Up on the hill where Menga is located, intense human activity also occurred during the first centuries of the 4th millennium BC^4,33^ evidenced by radiocarbon dates on animal bone and charred material obtained from the dolmen’s mound and various pits around it^1,33^, as well as multiple kinds of material culture^33^.

The already-existing ritual landscape at Antequera received a remarkable boost when, between 3800 and 3600 BCE, Menga was built. This megalithic monument, conceived to be the largest construction of its time, has a total inner length of 24.9 m, with a maximum width of 5.7 m and a height rising from 2.5 m at the entrance to 3.45 m at the back of the chamber, and includes 12 stone uprights on each side plus a massive back stone, five capstones and three pillars. A fourth pillar, possibly located near the entrance, appears to be missing (Fig. S3). The Menga Dolmen is an outstanding construction weighing over 1140 tons in total^3^ and containing some of the largest stones found in any European megalithic monument of its time. The largest piece, capstone #5, weighs about 150 tons.

Previous geoarchaeological studies focused on the orthostats and pillars, showing its geometrically irregular design^32^. This is reflected in the weights, lengths, volumes and lithologies utilised on both sides of the dolmen along its longitudinal axis. The dolmen’s geometry and orientation are such that, during the summer solstice, the sun illuminates the imperfect right side, while the smooth curve of the left side provides uniform darkness, a feature that has been observed in other dolmens in southern Iberia^1^. The Menga hill is the only place that offers a view of the singular anthropomorphic silhouette of La Peña de los Enamorados and astronomical orientation to the summer solstice.

**Text S3. Lithological characterization of other similar materials from the Upper Tortonian**

On the backyard of the La Trinidad church, 400 m to the south-west of Cerro de la Cruz, there is a stratigraphic section (Fig. S4a-c), with materials at the base, typical of fan-delta, which is identical to those identified below Menga and Viera, Los Remedios neighbourhood and Cerro de la Cruz. It is followed by a discordant erosive base, with typical materials of a submarine canyon, but in this case of lobes. This is characterised by a quartz-grain rich bioclastic calcirudite and includes algo a large amount of bivalves and, to a lesser extent, bryozoans. This material is about 1 to 1.20 m thick and it is cut by highly penetrative fractures of metric scale (Fig. S4b, c). Above the lobes, shoals materials similar to those found at Cerro de la Cruz and Los Remedios neighbourhood are deposited.

The factory zone facies are only found on the north-east side of El Hacho hill (Fig. S4a), in a small but highly homogeneous rocky outcrop about 2 m deep. The subfacies visible there include coralline red algae (Fig. S4d, e), with sizes up to 5 cm and, to a lesser extent, the nodular bryozoan-bivalves subfacies. These factory materials are not as affected by tectonic fracturing as those at the La Trinidad church section.

**Text S4. Calculation of the approximate weight of capstone C 5**

The weight of capstone C 5 was estimated as follows:

The surface area of the upper side was estimated from the drawing in figure 3 using Fiji software^34^ (see figure S5), yielding 41.024m^2^.

Capstone C 5 appears flat on the inner side of the dolmen but the upper side was found to be convex during a prospective excavation carried out by University of Malaga in 1991 (see sketch S6). Capstone C 5 is 1.83 m at its thickest point^3^ but was estimated to have an average height of approximately 1.63 m. This results in a total volume of 41.024m^2^ x 1.63 m = 66.87 m^3^. Considering the apparent density of the source calcirudite/micro-breccia (2237 kg/m^3^) capstone C 5 weights approximately 149.59 tons. Given the nature of our field measurements, our height estimate may vary by at most ± 10 cm in the worst-case scenario. Thus, we conclude that capstone C 5 is about 149.59 ± 9.17 tons.

**References**

1. Lozano, J. A., Ruiz-Puertas, G., Hódar-Correa, M., Pérez-Valera, F. & Morgado, A. Prehistoric engineering and astronomy of the great Menga Dolmen (Málaga, Spain). A geometric and geoarchaeological analysis. *Journal of Archaeological Science* **41**, 759–771 (2014).

2. National Geographic Information Center of Spain (IGN). Centro de Descargas del CNIG (IGN). *Centro de Descargas del CNIG* http://centrodedescargas.cnig.es.

3. Carrión Méndez, F. *et al.* *Estudio geoarqueológico de los sepulcros megalíticos de cueva de Menga, Viera y Romeral (Antequera, Málaga).* 219 (2006).

4. García Sanjuán, L. G. *et al.* In the bosom of the Earth: a new megalithic monument at the Antequera World Heritage Site. *Antiquity* **97**, 576–595 (2023).

5. García-Hernández, M., López-Garrido, A. C., Rivas, P., Sanz de Galdeano, C. & Vera, J. A. Mesozoic palaeogeographic evolution of the External Zones of the Betic Cordillera. (1980).

6. Sanz de Galdeano, C. *La zona interna bético-rifeña: (antecedentes, unidades tectónicas, correlaciones y bosquejo de reconstrucción paleogeográfica)*. (Universidad de Granada, 1997).

7. Vera, J. A. Evolution of the South Iberian Continental Margin. In *Peri-tethyan rift/wrench basins and passive margins. (eds. Ziegler, P. A., Cavazza, W., Robertson, A. H. F.& Crasquin-Soleau, S.) (Mémoires du Muséum national d’histoire naturelle. Paris, 2001).* 109–143 (2001).

8. Martín-Algarra, A. & Vera, J. A. Divisiones mayores y nomenclatura. In *Geología de España, SGE-IGM, Madrid* (ed. Vera, J. A.) 348–350 (2004).

9. Vera, J. A. & Molina, J. M. La formación Capas Rojas: caracterización y génesis. *Estudios Geológicos* **55**, 45–66 (1999).

10. Pérez-Valera, F., Sánchez-Gómez, M., Pérez-López, A. & Pérez-Valera, L. A. An evaporite-bearing accretionary complex in the northern front of the Betic-Rif orogen. *Tectonics* **36**, 1006–1036 (2017).

11. Sanz de Galdeano, C., Lozano Rodríguez, J. A. & Puga, E. EL «Trías de Antequera»: Naturaleza, Origen y Estructura. *Revista de la Sociedad Geológica de España* **21**, 111–124 (2008).

12. Pérez-Valera, F., Sánchez-Gómez, M. & Pérez-Valera, L. A. From salt diapirs to strike-slip tectonic push-up structures: Outcropping examples from the Triassic evaporites of the Betic Cordillera (south-east Spain). MAPG-AAPG 2nd International Convention, Conference and Exhibition, Marrakech. (2011).

13. Puga-Bernabéu, Á., Martín, J. M. & Braga, J. C. Sedimentary processes in a submarine canyon excavated into a temperate-carbonate ramp (Granada Basin, southern Spain). *Sedimentology* **55**, 1449–1466 (2008).

14. Serrano, F. *Los foraminiferos plantonicos del Mioceno Superior de la Cuenca de Ronda y su coparación con las de otras áreas de las Cordilleras Béticas*. (Universidad de Málaga, 1979).

15. Jiménez Aranda, G. A. *et al.* La cronología radiocarbónica de las primeras manifestaciones megalíticas en el sureste de la Península Ibérica: las necrópolis de Las Churuletas, La Atalaya y Llano del Jautón (Purchena, Almería). *Trabajos de Prehistoria* **74**, 257–277 (2017).

16. Lozano Medina, Á. & Aranda Jiménez, G. Long-lasting sacred landscapes: The numerical chronology of the megalithic phenomenon in south-eastern Iberia. *Journal of Archaeological Science: Reports* **19**, 224–238 (2018).

17. Linares-Catela, J. A. Radiocarbon Chronology of Dolmens in the Iberian Southwest: Architectural Sequence and Temporality in the El Pozuelo Megalithic Complex (Huelva, Spain). *Radiocarbon* **64**, 989–1064 (2022).

18. Vijande-Vila, E. *et al.* At the beginnings of the funerary Megalithism in Iberia at Campo de Hockey necropolis. *Sci Rep* **12**, 9431 (2022).

19. Márquez-Romero, J. E. & Jiménez-Jáimez, V. Monumental ditched enclosures in southern Iberia (fourth–third millennia BC). *Antiquity* **87**, 447–460 (2013).

20. Márquez Romero, J. E. & Jiménez Jáimez, V. *Recintos de fosos: genealogía y significado de una tradición en la Prehistoria del suroeste de la Península Ibérica (IV-III milenios AC)*. (Servicio de Publicaciones e Intercambio Científico, 2010).

21. Cruz Berrocal, M. C., Sanjuán, L. G. & Guillén, A. G. *The Prehistory of Iberia: Debating Early Social Stratification and the State*. (Routledge Reino Unido, 2013).

22. Morgado Rodríguez, A. & Roncal Los Arcos, M. E. *Los últimos talladores del sílex: estudio histórico-arqueológico sobre la explotación del sílex en las tierras de Loja y la producción militar de piedras de chispa del reino de Granada durante los siglos XVIII y XIX*. (Fundación Ibn al-Jatib de Estudios de Cooperación Cultural, 2009).

23. Morgado-Rodríguez, A. & Lozano Rodríguez, J. A. The Impact of Geological Factors on Flint Mining and Large Blade Production in the Betic Cordillera (Spain, 4th – 3rd mill. BC). *In Proceedings of the 2nd International Conference of the UISPP Commission on Flint Mining in Pre-and Protohistoric Times BAR International Series (eds. Capote, M., Consuegra, S., Díaz-del-Río, P. & Terradas. (British Archaeological Reports International Series* 182–191 (2011).

24. Lozano, J. A., Morgado Rodríguez, A., Puga Rodríguez, E. & Martín Algarra, A. Explotaciones de sílex tipo ‘Turón’ (Málaga, España): localización y caracterización petrológica y geoquímica. *Geogaceta* 163–166 (2010).

25. Rodríguez-Tovar, F. J., Morgado, A. & Lozano, J. A. Using ichnofossils to characterize chert tools: A preliminary study from Southern Iberia. *Geoarchaeology* **25**, 514–526 (2010).

26. Rodríguez-Tovar, F. J., Morgado, A. & Lozano, J. A. Ichnological analysis: a non-destructive tool in archaeology. *Lethaia* **43**, 587–590 (2010).

27. Fernández Rodríguez, L. E. & Rodríguez Vinceiro, F. J. La explotación de recursos minerometalúrgico cupríferos durante la Prehistoria reciente en el Bético de Málaga. In *Los recursos abióticos en la prehistoria: caracterización, aprovisionamiento e intercambio, (eds. Bernabeu, J., Orozco, T. & Terradas, X.)* 155–172 (Servicio de Publicaciones = Servei de Publicacions, 1998).

28. Manrique, J. T. & Rodríguez, A. M. El aprovechamiento Prehistórico de sal en la Alta Andalucía. El caso de Fuente Camacho (Loja, Granada). *Cuadernos de Prehistoria y Arqueología de la Universidad de Granada* **21**, 221–249 (2011).

29. Martínez-Sevilla, F., Carrasco Rus, J., Lozano Rodríguez, J. A., Jiménez-Cobos, F. & Gutiérrez Rodríguez, M. Un sitio de extracción de mármol para hacer brazaletes: la cantera neolítica de Cortijo Cevico (Ventorros de San José- Loja, Granada). *Trab. prehist.* **75**, 344 (2018).

30. Martínez-Sevilla, F., Piper, S. F., Jiménez Cobos, F., Lozano Rodríguez, J. A. & Carrasco Rus, J. Shaping Marble, Shaping Minds: Apprenticeship in an Early Neolithic Bracelet Quarry. *Lithic Technology* **45**, 1–18 (2020).

31. Martínez-Sevilla, F., Lozano Rodríguez, J. A. & Morgado, A. The transformations of ochre in the late Neolithic of Granada Depression: tools and their use-wear. International Conference on use-wear analisys. Use-wear 2012. (Universidade do Algarve). (2012).

32. García Sanjuán, L. *et al.* Builders of Megaliths: Society, monumentality and environment in 4th millennium cal BC Antequera. *Journal of Archaeological Science: Reports* **33**, 102555 (2020).

33. García Sanjuán, L. *et al.* A multimethod approach to the genesis of Menga, a World Heritage megalith. *Quat. res.* **111**, 1–20 (2023).

34. Schindelin, J. *et al.* Fiji: an open-source platform for biological-image analysis. *Nat Methods* **9**, 676–682 (2012).
